# Evaluating single-best-answer questions in dental materials education: a retrospective item analysis from an online undergraduate dentistry question bank

**DOI:** 10.1038/s41405-026-00481-6

**Published:** 2026-09-28

**Authors:** Oliver Vij, Alannah Herbert, Joshua Penning-Lambert, Tyler Longbone, Sanjukta Deb

**Affiliations:** 1 Faculty of Dentistry, Oral and Craniofacial Sciences, King’s College London, Guy’s Hospital, London, UK; 2 Lucy Cavendish College, University of Cambridge, Cambridge, UK; 3 Walsall Manor Hospital, Walsall Healthcare NHS Trust, London, UK

**Keywords:** Dental education, Dental materials

## Abstract

**Background:**

High-quality single-best-answer (SBA) questions require appropriate construction, alignment with intended learning outcomes, absence of technical item flaws and psychometric evaluation. Dentistry-specific evidence linking these constructional features to item performance remains limited. This study analysed Dental Materials responses from DentTest, an online undergraduate dentistry question bank, to identify item characteristics associated with higher discrimination.

**Methods:**

A retrospective learning-analytics item analysis was performed using first-attempt response data. At extraction, 134 users were eligible, of whom 76 provided research consent (56.7%). The dataset contained 44,964 user×item observations from 76 users across 1560 Dental Materials items. Primary analyses were restricted to 691 items with ≥30 unique first-attempt users. Analyses included item psychometric parameters (facility, discrimination index, number of functioning distractors and distractor entropy) and selected constructional features (option-length cueing, stem word count and negative-stem wording).

**Results:**

Discrimination index was inversely correlated with facility (*r* = −0.471; 95% CI –0.527 to –0.411). In the multivariable quadratic model, the estimated peak discrimination occurred at *p* = 0.578, consistent with an inverted-U relationship between facility and discrimination. Functioning distractors (*r* = 0.531; 95% CI 0.475–0.582) and distractor entropy (*r* = 0.471; 95% CI 0.411–0.527) were positively associated with discrimination index. However, split-half stability was low (*r* = 0.15), with 542/691 items (78.4%) changing discrimination band at least once across repeated splits.

**Conclusion:**

In this formative dentistry question bank, Dental Materials SBAs with higher discrimination were associated with moderate facility, stronger distractor functioning and more informative distractor-choice patterns. Item analytics should be viewed as an early-warning system rather than a final judgement, guiding educators towards items that require revision.

## Introduction

Online question banks are now a routine part of revision culture across healthcare education, with single-best-answer (SBA; Type A multiple-choice) formats widely used for formative and summative assessment [1, 2]. However, the educational value of a question bank depends not only on content coverage but also on the quality of individual items, specifically, whether questions can meaningfully distinguish between learners of different ability and whether distractors function as plausible alternatives rather than obvious “throwaway” options. Established item-writing guidance in the health sciences (e.g., NBME manuals) and evidence syntheses emphasise avoiding technical item flaws and writing options that are plausible, homogeneous, and free from cueing [3, 4].

A core psychometric indicator of item quality is the discrimination index, which reflects how well an item differentiates higher-performing from lower-performing examinees [5]. The classical upper-lower group approach calculated discrimination as the difference between the proportion correct in top versus bottom groups; Kelley’s early work suggested selecting extreme groups (often cited as the upper and lower 27%) to maximise discrimination under certain assumptions, although practical implementations often adapt group sizes to available response counts [5, 6].

In addition to discrimination, the performance of distractors is central to item quality. A substantial literature in medical and nursing education has demonstrated that many distractors in five-option multiple-choice questions (MCQs) are non-functioning [5]. A non-functioning option is commonly operationalised as one selected by fewer than 5% of examinees, and distractor functioning is associated with item discrimination and overall test efficiency [7]. To contextualise the study, we searched PubMed, Google Scholar and reference lists for dental education studies across the following: dentistry; dental materials; oral medicine; prosthodontics; endodontics. Relevant dental-specific studies were limited, and most available evidence came from medical, nursing or general educational assessment literature. This evidence gap is especially marked in specialist foundation subjects such as Dental Materials. Most published studies focus on exam question analyses from individual institutions rather than large-scale analyses of formative question banks [8]. Dental Materials assessment often requires learners to integrate concepts spanning structure-property relationships, setting reactions, clinical indications and failure modes. This creates a strong rationale for empirically testing which item features, such as facility, distractor plausibility patterns and potential cueing from option length, are associated with higher discrimination in authentic use.

The growth of digital learning platforms enables scalable “learning analytics” item evaluation using real-world response data. In addition to traditional metrics such as item facility and discrimination, digital logs enable analysis of distractor choice distributions, including the number of functioning distractors and the spread of incorrect selections, which can be quantified using information-theoretic measures such as Shannon entropy [9]. In this context, distractor (Shannon) entropy describes the evenness of spread of incorrect responses across the available distractors: low entropy indicates that incorrect responses are concentrated on one distractor, whereas high entropy indicates that responses are distributed more evenly across several distractors.

The aim of this study was to use first-attempt response data from the online undergraduate question bank DentTest to:

1. Describe distributions of item facility and discrimination index in Dental Materials SBAs;
2. Quantify the association between discrimination index, distractor functioning and distractor choice distributions;
3. Evaluate whether option-length cueing metrics are associated with discrimination index after accounting for facility and distractor quality.

By linking item analytics to actionable writing features, we sought to provide a data-informed heuristic for prioritising item review and revision, rather than a definitive rule set for item quality.

## Materials and methods

### Study design and setting

We performed a retrospective learning-analytics item analysis of Dental Materials single-best-answer (SBA) questions hosted on DentTest.co.uk (DentTest - Undergraduate Dentistry Question Bank & Textbook), an online undergraduate dentistry question bank.

### Ethical approval and consent

Ethical clearance was granted by King’s College London (registration confirmation reference is MRSU-25/26-56180). Analyses were restricted to users who provided explicit consent for research use of their data. All datasets were pseudonymised before analysis, and data processing was registered with King’s College London in accordance with UK GDPR requirements.

### Data sources and consent filtering

Three exports (generated on 11th June 2026) were used:

1. User×item first-attempt dataset: one row per consented user_id × question_id containing the earliest attempt timestamp, first-attempt correctness (0/1), and option selected (A–E).
2. Item metadata dataset: one row per question containing stem and option text, stem word count, negative-stem flag, and option character-length cueing metrics.
3. User ability summary dataset: per-user Dental Materials first-attempt accuracy (used descriptively; primary analyses recomputed directly from the user×item dataset).

### Definitions

First attempt:

The first attempt for each user–question pair was defined as the earliest recorded attempt timestamp for that user on that question. Only first attempts were analysed to reduce bias due to repeat attempts and memorisation.

Item facility:

Facility was defined as the proportion of first attempts answered correctly:

$$p=\frac{{correct\; first\; attempts}}{{total\; first\; attempts}}$$

Leave-one-item-out Dental Materials ability:

To rank users for discrimination estimation while minimising part–whole contamination, we computed a leave-one-item-out (LOO) Dental Materials ability score for each user *u* and index item *i*:

$${Ability}_{DM}\left(-i\right)\left(u\right)=\frac{{\sum }_{j\ne i}{\mathbb{1}}({{correct}}_{u,j})}{{\sum }_{j\ne i}{\mathbb{1}}({{attempted}}_{u,j})}$$

Where *j* indexes Dental Materials items attempted by user *u* on first attempt. Users with fewer than two Dental Materials items (after excluding the index item) were excluded for that item’s discrimination grouping. This criterion affected 2 of 44,964 user×item pairs (0.0044%), involving one user who had attempted only two Dental Materials items. Neither affected item met the ≥30-user threshold for the primary analytic set; therefore, no user×item pairs in the primary analysis were excluded on this basis.

### Discrimination index (upper–lower tertiles)

For each item, eligible users who attempted the item (first attempts only) were ranked by ${{Ability}}_{{DM}\left(-i\right)}.\,$ The top tertile and bottom tertile were defined using group size [N/3], where N is the number of eligible users for that item (middle tertile discarded). Although Kelley’s original approach used upper and lower 27% groups under specific assumptions, we used tertiles to retain slightly larger comparison groups at the observed response volumes [6]. This choice was pragmatic and intended to reduce, but not eliminate, sampling variability in the small consenting cohort.

Discrimination index (*D*) was calculated as:

$$D={p}_{{top}}-{p}_{{bottom}}$$

Discrimination bands were predefined as: Excellent (D ≥ 0.40), Good (0.30 ≤ D < 0.40), Fair (0.20 ≤ D < 0.30), Poor (0.00 ≤ D < 0.20), and Problematic (D < 0).

### Distractor and cueing metrics

Using first-attempt option selections, we computed item-level distractor behaviour:

- Functioning distractors ( ≥ 5%): number of incorrect options selected by ≥5% of users on first attempt [5].
- Distractor entropy: Shannon entropy of the incorrect-option selection probabilities (renormalised across distractors), quantifying how evenly incorrect selections were distributed [9]. Entropy was computed across incorrect options only, with incorrect-option probabilities renormalised to sum to 1 for each item.
- Option-length cueing: absolute correct-option character-length z-score |z| (from item metadata), indicating whether the keyed option is unusually long/short relative to the other options.
- Correct/median length ratio: the correct option’s character count divided by the median character count of all options.

Cueing analysis was restricted to option character-length metrics. Other technical item flaws, including grammatical cueing, absolute terms, convergence cues, repeated wording between stem and key, “all/none of the above” options, irrelevant difficulty and cover-the-options performance, were not automatically coded in this analysis. These features were therefore treated as outside the scope of the quantitative cueing metric.

### Primary analytic set

Given the consented cohort size, we defined the primary analytic set a priori as items with ≥30 unique consented first-attempt users. This threshold ensured that the top and bottom tertiles typically contained at least 10 users each, allowing estimation of *p*_*top*_ and *p*_*bottom*_ without extremely small group sizes. We note that discrimination estimates at this response volume may still be unstable and, therefore, quantified stability empirically using split-half procedures. The consenting cohort was small for item-analysis purposes, particularly for estimating stable discrimination indices. The ≥30-user threshold was therefore chosen to ensure minimum group sizes for exploratory, item-level review rather than to imply that item statistics were stable enough for high-stakes interpretation [10].

### Statistical analysis

Analyses were performed at the item level. Continuous variables are reported as mean (SD) or median (IQR) as appropriate. Associations between discrimination and predictors were summarised using Pearson correlation coefficients.

To estimate independent associations with discrimination, we fitted multivariable linear regression models with *D* as the dependent variable. Models included facility *p* and *p*^*2*^ (to capture the expected non-linear relationship), functioning distractors, distractor entropy, option-length cueing ( | z | ), stem word count, and negative-stem flag. Inference used heteroskedasticity-robust (HC3) standard errors. For items in which all answer options had identical character lengths, the within-item standard deviation was zero and the z-score was mathematically undefined; these items were assigned an absolute length-deviation score of 0 because the keyed option did not differ in length from the distractors.

To quantify the stability of discrimination estimates at the observed response volumes, we performed a split-half stability analysis on items in the primary analytic set. For each item, eligible first-attempt users were randomly split into two approximately equal halves; discrimination *D* was computed separately in each half using the same tertile procedure, and agreement was summarised by the split-half correlation and mean absolute split-half difference. We additionally estimated the proportion of items that changed discrimination band across repeated random splits. This procedure was repeated 50 times per item to assess discrimination-band stability.

All analyses were conducted using Python (pandas/statsmodels).

## Results

### Sample and item coverage

At the time of data extraction, 134 users were eligible for inclusion, of whom 76 (56.7%) had provided research consent and were included. The consent-filtered first-attempt dataset contained 44,964 user×item observations from 76 unique users across 1560 Dental Materials items. Users attempted a mean of 591.6 Dental Materials items each on first attempt. Across the full pool of 1560 Dental Materials items, the mean number of consented first-attempt users per item was 28.8.

### Primary analytic set characteristics ( ≥ 30 users per item)

The primary analytic set comprised 691 items with ≥30 users; the median number of unique first-attempt users per item was 33 (IQR 31–35). Mean facility was *p* = 0.825 ± 0.167, indicating that most items were relatively easy in this cohort. Mean discrimination was *D* = 0.166 ± 0.169. A banded summary is provided in Table 1.

**Table 1:** Dental Materials item characteristics by discrimination band.

Discrimination index band	Items, n	Users per item, median [IQR]	Facility (proportion correct on first attempt), mean ± SD	Discrimination index D, mean ± SD	Functioning distractors ( ≥ 5%), mean ± SD	Distractor entropy, mean ± SD	Correct-option length deviation (absolute z-score), mean ± SD	Correct/median length ratio, mean ± SD
Overall (consented; n ≥ 30 users)	691	33 [31–35]	0.825 ± 0.167	0.166 ± 0.169	1.15 ± 1.05	0.529 ± 0.453	0.967 ± 0.505	1.086 ± 0.291
Problematic (D < 0)	29	33 [31–36]	0.791 ± 0.229	-0.096 ± 0.029	1.28 ± 1.13	0.392 ± 0.476	1.059 ± 0.435	1.040 ± 0.232
Poor (0 ≤ D < 0.20)	428	33 [31–35]	0.883 ± 0.141	0.078 ± 0.071	0.74 ± 0.89	0.384 ± 0.434	0.992 ± 0.513	1.108 ± 0.289
Fair (0.20 ≤ D < 0.30)	89	34 [32–36]	0.763 ± 0.150	0.249 ± 0.025	1.69 ± 0.87	0.782 ± 0.347	0.855 ± 0.476	1.046 ± 0.290
Good (0.30 ≤ D < 0.40)	70	33 [31–36]	0.745 ± 0.134	0.340 ± 0.028	1.71 ± 0.85	0.783 ± 0.353	0.927 ± 0.491	1.070 ± 0.277
Excellent (D ≥ 0.40)	75	33 [30–35]	0.653 ± 0.141	0.507 ± 0.099	2.31 ± 0.80	0.870 ± 0.309	0.958 ± 0.521	1.046 ± 0.326

Discrimination bands are presented descriptively and should be interpreted cautiously because individual-item D estimates showed low split-half stability at the observed response counts.

Given the high mean facility (*p* = 0.825 ± 0.167), the large proportion of items in the poor discrimination band (428/691; 61.9%) should not be interpreted solely as evidence of poor item construction. For very easy items, the maximum possible separation between upper- and lower-performing users is constrained because most users answer correctly. The poor-discrimination band therefore reflects both item quality and the ceiling effect created by high facility in this cohort.

### Discrimination stability

Split-half analysis demonstrated low stability of discrimination estimates at current response volumes. Across items in the primary analytic set, split-half *D* values were weakly correlated (*r* = 0.15) with a mean absolute split-half difference of 0.204. Across 50 repeated random splits per item, 542/691 items (78.4%) changed discrimination band at least once. These findings indicate that discrimination index values were not reliable enough to classify individual items definitively at the observed response counts. Accordingly, *D* should be interpreted as a provisional item-review signal rather than a stable item property.

### Facility–discrimination relationship

**Fig. 1:**
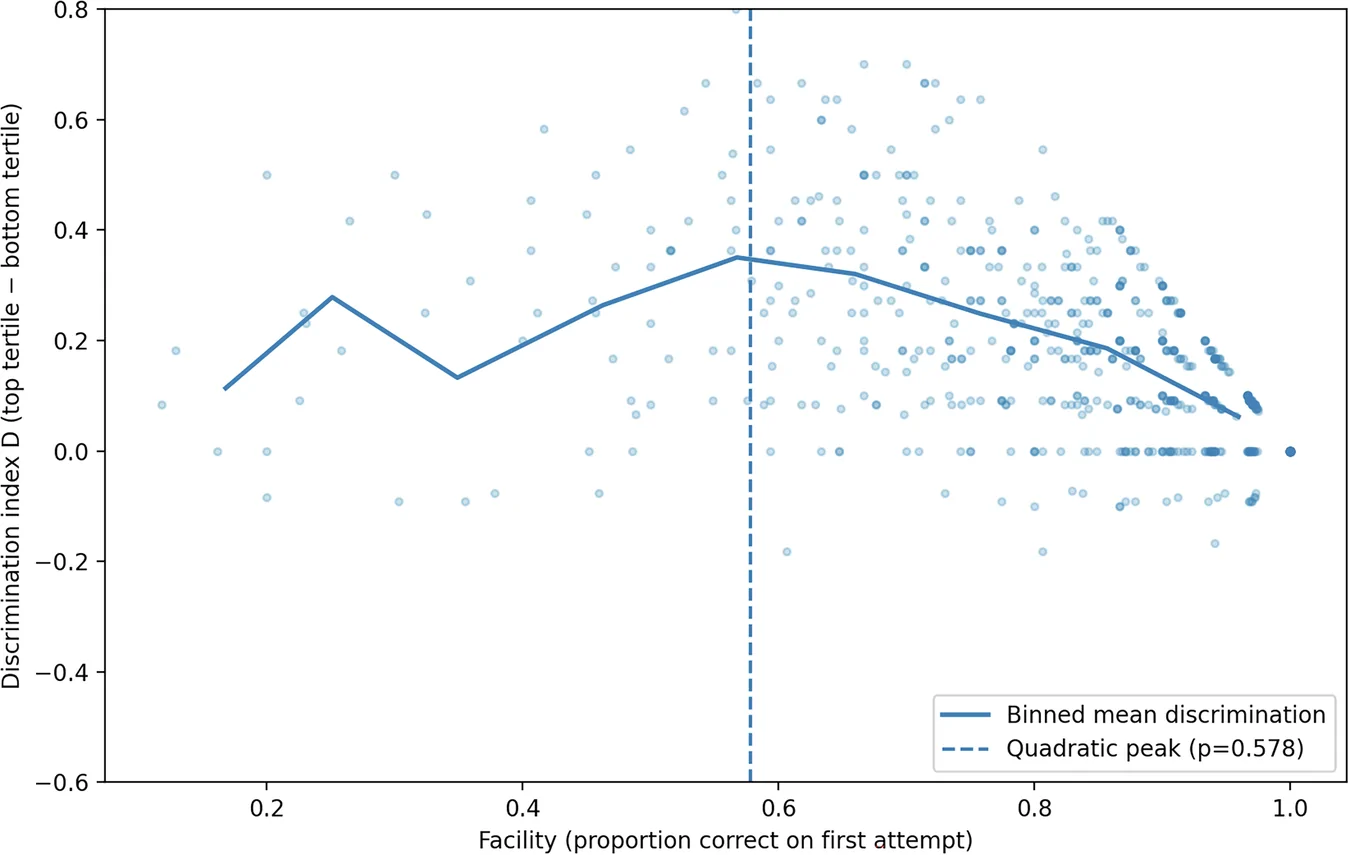
Relationship between facility (proportion correct on first attempt) and discrimination index.

Discrimination index (*D*) was inversely correlated with facility (proportion correct on first attempt) (*r* = −0.471; 95% CI –0.527 to –0.411). Visual inspection demonstrated a non-linear relationship in which discrimination peaked at moderate facility and declined for very easy items (Fig. 1). In the multivariable quadratic model, the estimated peak discrimination occurred at *p* = 0.578, consistent with an inverted-U relationship between facility and discrimination.

### Distractor functioning and discrimination

Items demonstrated a strong positive association between discrimination and distractor functioning. Functioning distractors ( ≥ 5%) were positively associated with discrimination (*r* = 0.531; 95% CI 0.475–0.582).

**Fig. 2:**
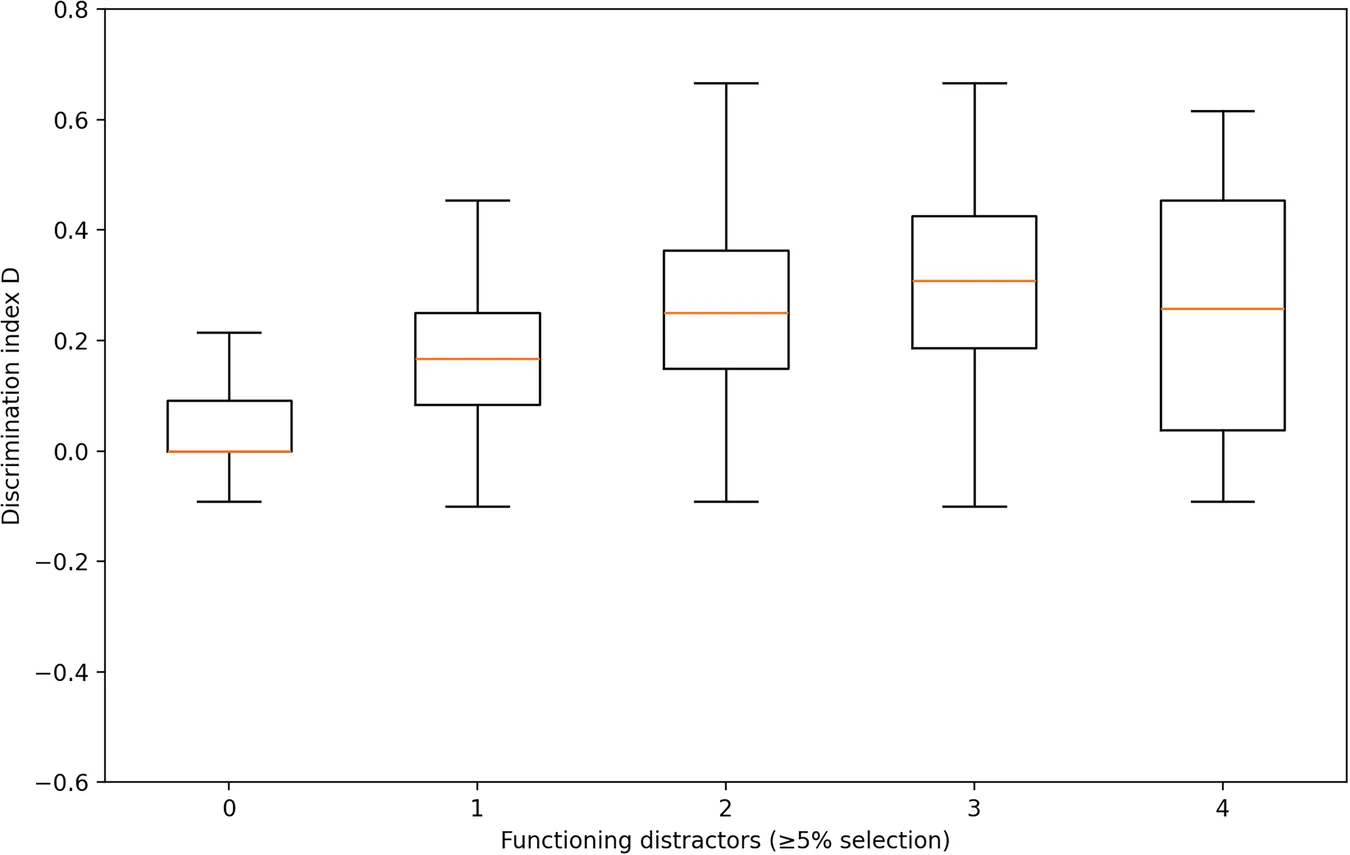
Discrimination index by number of functioning distractors.

Median discrimination increased with the number of functioning distractors (Fig. 2), indicating that items with more plausible distractors were more effective at differentiating higher- and lower-performing users within this cohort.

### Distractor entropy and discrimination

**Fig. 3:**
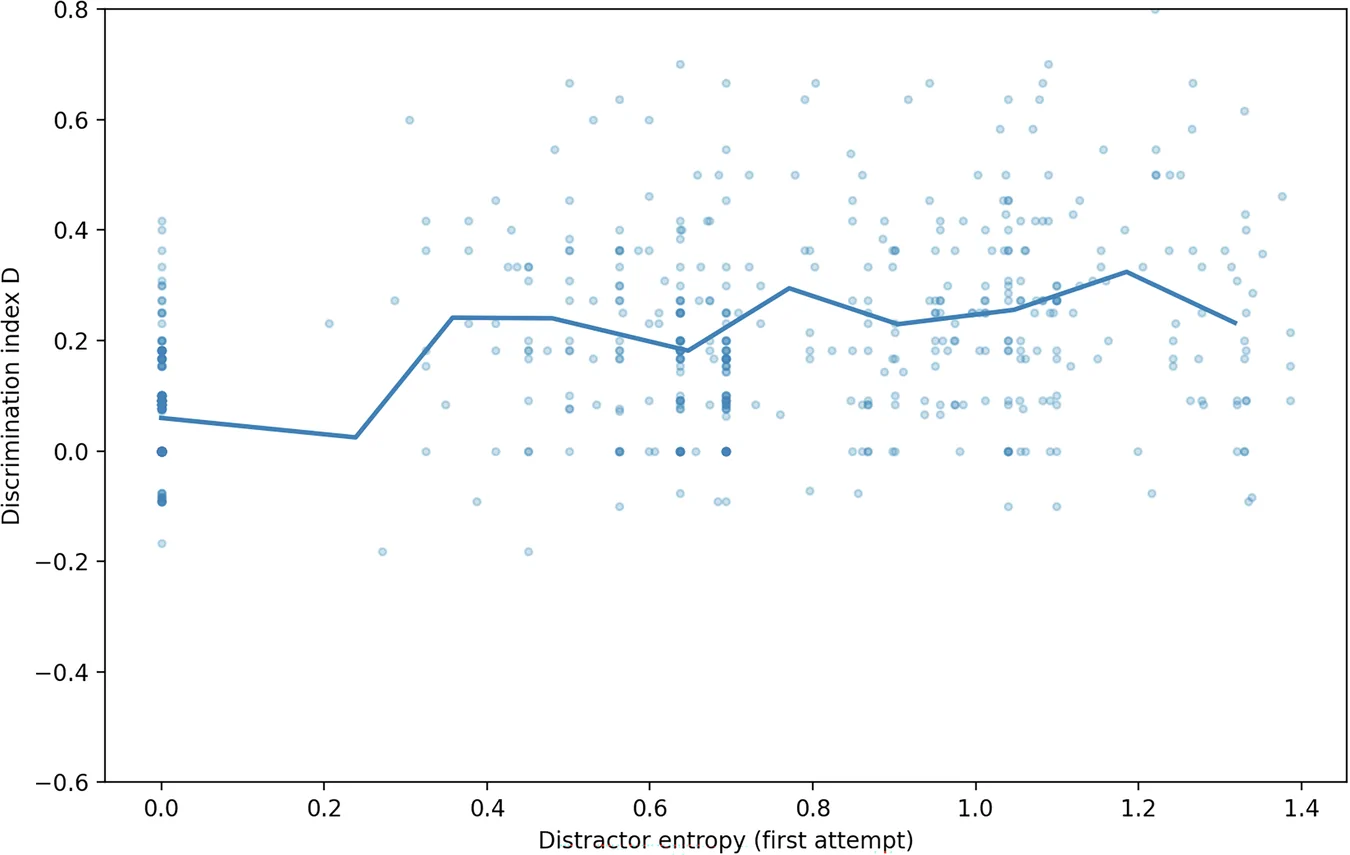
Relationship between distractor entropy and discrimination index.

Distractor entropy was positively associated with discrimination (*r* = 0.471; 95% CI 0.411–0.527). Items with higher entropy (more even distribution of incorrect selections across distractors) tended to show higher discrimination (Fig. 3).

### Option-length cueing and discrimination

**Fig. 4:**
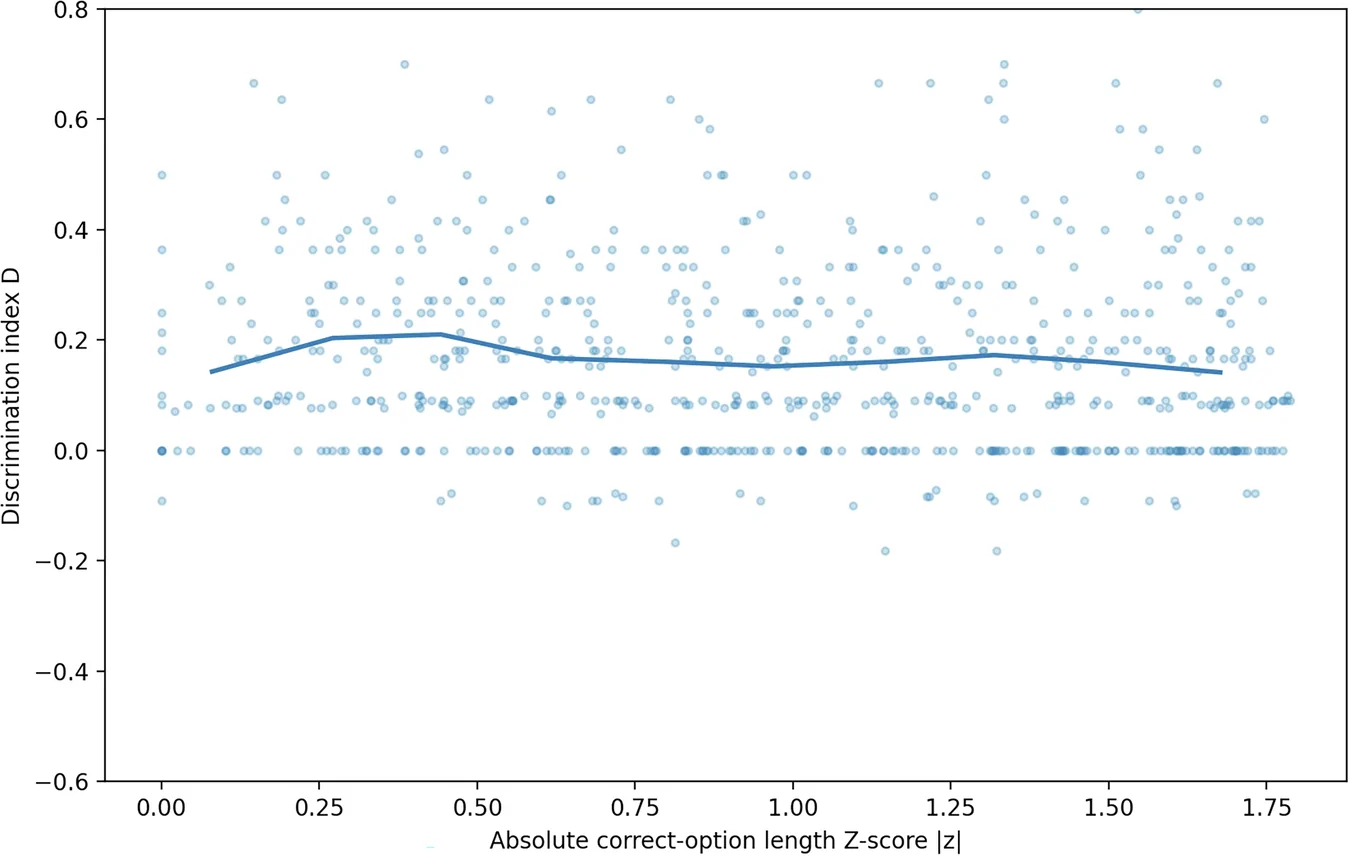
Relationship between correct-option length deviation and discrimination index.

Option-length cueing, operationalised as the absolute correct-option character-length z-score |z | , showed minimal association with discrimination (*r* = −0.066; 95% CI –0.140 to 0.008). The relationship is shown in Fig. 4.

Given the low split-half stability of *D*, these correlations should be interpreted as aggregate item-level patterns across the 691-item analytic set, rather than as precise evidence about any individual item.

### Multivariable model

The full multivariable regression model is presented in Table 2. The model included all 691 items in the primary analytic set and explained 38.4% of the observed variance in discrimination index (*R*² = 0.384; adjusted *R*² = 0.378; HC3 robust F = 59.64, *p* < 0.001). Facility showed a significant non-linear association with discrimination index, with a positive linear facility term (*β* = 1.523, 95% CI 1.038–2.008; *p* < 0.001) and negative quadratic term (*β* = −1.317, 95% CI − 1.687 to −0.947; *p* < 0.001), consistent with an inverted-U relationship. After adjustment for facility and the other included item characteristics, distractor entropy remained positively associated with discrimination index (*β* = 0.0360, 95% CI 0.0075–0.0645; *p* = 0.013). The number of functioning distractors showed a positive but statistically non-significant association (*β* = 0.0190, 95% CI − 0.0021 to 0.0401; *p* = 0.078). Correct-option length deviation, stem word count and negative-stem wording were not independently associated with discrimination index.

**Table 2:** Multivariable linear regression of item characteristics associated with discrimination index (D).

Predictor	*β*	HC3 robust SE	95% confidence interval	*p* value
Intercept	–0.1958	0.0839	–0.3602 to –0.0313	0.020
Facility, p	1.5232	0.2475	1.0381 to 2.0083	<0.001
Facility^2^, p^2^	–1.3172	0.1887	–1.6871 to –0.9473	<0.001
Functioning distractors ( ≥ 5%)	0.0190	0.0108	–0.0021 to 0.0401	0.078
Distractor entropy	0.0360	0.0145	0.0075 to 0.0645	0.013
Correct-option length deviation (absolute z-score)	0.0026	0.0106	–0.0182 to 0.0233	0.809
Stem word count	–0.00023	0.00039	–0.00099 to 0.00053	0.554
Negative-stem flag	–0.0035	0.0277	–0.0578 to 0.0509	0.900

D was the dependent variable. β values are unstandardised regression coefficients. Standard errors and confidence intervals are heteroskedasticity-robust (HC3).

In sensitivity analyses restricted to items with ≥35 users per item (*n* = 227), coefficient directions were similar, but confidence intervals widened, and associations were less precise due to reduced sample size.

## Discussion

In this item-analytics study of an undergraduate Dental Materials SBA bank, we identified item features associated with higher discrimination in this formative, self-directed online setting, using first-attempt responses and subject-specific performer ranking with a leave-one-item-out ability score. These findings describe within-platform item behaviour and should not be assumed to generalise directly to summative examinations without further validation. These findings align with assessment guidance that exam quality and fairness depend not only on avoiding technical item flaws, but also on constructive alignment with intended learning outcomes, appropriate blueprinting, plausible option construction and post-hoc psychometric review [3, 4]. The formative context is central to interpretation; DentTest is used for self-directed practice and feedback, so item analytics are most appropriately viewed as tools for iterative item improvement and learning support rather than as evidence for high-stakes decision-making.

Three findings were most salient. Firstly, discrimination showed a clear non-linear relationship with facility, with the highest discrimination occurring at moderate facility (*p* = 0.578). Second, distractor quality was associated with higher discrimination at the univariable level. In the multivariable model, distractor entropy remained independently associated with discrimination, whereas the number of functioning distractors retained a positive direction of association but did not meet conventional statistical significance after adjustment. Third, option-length cueing showed only a weak relationship with discrimination and did not remain an independent predictor once facility and distractor metrics were included; nevertheless, it remains a useful quality-control flag when refining items.

The low split-half stability of *D* materially qualifies these findings. At the individual-item level, *D* was unstable and should not be used as a definitive classification of item quality at current response counts. However, the correlation and regression analyses were performed across 691 items and may still identify aggregate item-level patterns, even when individual item estimates are noisy. We therefore interpret the observed associations between facility, distractor functioning, entropy and *D* as provisional signals for item-review prioritisation rather than precise estimates of stable item properties.

The observed inverted-U relationship between facility and discrimination is consistent with long-standing assessment theory: extremely easy items have limited capacity to separate higher- and lower-performing learners because most users answer correctly, whereas very hard items may elicit guessing [11]. Empirical item-analysis studies commonly demonstrate that extreme facility (very easy or very hard items) is associated with reduced discrimination, supporting the observed non-linear relationship [12, 13]. In practice, this means that “good” Dental Materials questions are not necessarily those with the highest proportion correct, but those that create a meaningful gradient of performance between learners. In our dataset, most items were relatively easy, which likely contributed to the predominance of items classified as “poor” discrimination index.

Distractor quality is a central determinant of MCQ/SBA measurement performance: non-functioning distractors are common in health-professions assessments (often operationalised as <5% selection), and increasing distractor plausibility is associated with improved discrimination and test efficiency [5, 14]. Items with higher discrimination had substantially more functioning distractors and more informative patterns of incorrect choice behaviour [15]. However, after adjustment for facility and other item characteristics, the association with functioning distractor count was attenuated and no longer statistically significant, whereas distractor entropy remained independently associated with discrimination index. In Dental Materials, these distractors may reflect common misunderstandings (e.g., confusing setting reactions, mixing material classes, misapplying structure–property relationships, or conflating clinical indications), but confirming this would require qualitative coding or expert review of distractor content. This is consistent with a design approach in which distractors are deliberately written to represent distinct, realistic misconceptions rather than generic wrong statements [16, 17]. Future work should validate misconception categories through expert coding of distractors and assessment of inter-rater agreement.

The Dental Materials context gives these findings particular practical relevance. Unlike items based primarily on factual recall, Dental Materials questions frequently require learners to distinguish between closely related materials, mechanisms, properties and clinical indications, where superficially plausible alternatives may differ by only one critical feature. Our findings suggest that this conceptual structure can be exploited when constructing distractors: alternatives based on competing material classes, incorrect structure–property relationships, inappropriate clinical indications or correct mechanisms applied under the wrong conditions may provide more informative distractors than generic incorrect statements. Although the present analysis did not formally classify distractor misconceptions, it provides empirical evidence from authentic Dental Materials question-bank use that distractor behaviour is an important target for subject-specific item review.

Technical item flaws can cue the correct answer and thereby distort item statistics; item-writing guidance explicitly warns against cueing patterns (including conspicuously long keyed options), and empirical work shows that flawed items can affect item performance [4, 18]. In this dataset, option-length metrics were weakly associated with discrimination and were not independent predictors in multivariable modelling. This suggests that length balance is best treated as a screening tool, useful for identifying items to review, rather than a primary determinant of discriminative performance. Importantly, the absence of a strong effect for option-length cueing does not mean cueing mechanisms are unimportant more broadly; we only operationalised cueing using character-length metrics, and did not assess other cueing mechanisms (e.g., grammatical cues, absolutes, patterning).

For formative question banks, item analytics are best understood as part of an iterative quality-assurance cycle. Facility, discrimination index, distractor functioning and cueing metrics can identify items that warrant review, but they should be interpreted alongside content accuracy, curriculum alignment and expert judgement.

Given the observed instability of *D* at current response counts, we present the following as prioritisation heuristics for item revision rather than definitive rules [3, 4]:

1. Aim for moderate facility (avoid extremely easy or extremely hard items)
  - High-discrimination items cluster around moderate facility. Items that are consistently answered correctly by >90% of users are unlikely to discriminate well unless distractors are exceptionally plausible. To improve discriminative value, shift from definitional recall to application (e.g., selecting a material given moisture control constraints, connector thickness, thermal expansion mismatch, surface treatment steps, or clinical failure scenarios) [3, 4, 11].
2. Write at least two functioning distractors
  - A practical target is at least two distractors each selected by ≥5% of first-attempt users. Items with 0–1 functioning distractors frequently showed poor discrimination. When an item performs poorly, revising distractors is a plausible first target, because items with more functioning distractors were associated with higher discrimination in this dataset [5, 7, 15]. Although this association was attenuated after multivariable adjustment, the strong unadjusted relationship supports retaining distractor review as a practical quality-assurance step.
  - This is consistent with evidence that additional distractors beyond three often fail to function and that reducing to three options can preserve psychometric quality when plausible distractors are difficult to write [14, 15].
3. Make distractors represent distinct misconceptions
  - Rather than writing “obviously wrong” options, use distractors that are close competitors:
    i. same material family but wrong subclass (e.g., resin-modified vs conventional glass ionomer),
    ii. correct property but wrong implication (e.g., high modulus vs toughness),
    iii. correct mechanism but wrong condition (e.g., moisture sensitivity at the wrong stage),
    iv. correct clinical indication but wrong surface treatment.
4. Prefer distractor sets that produce informative wrong-answer patterns
  - Higher entropy (a spread of incorrect selections across distractors) was associated with higher discrimination. Where functioning distractor counts indicate whether distractors attract learners, entropy additionally reflects whether incorrect selections are distributed across multiple plausible alternatives rather than collapsing onto a single distractor [5, 9].
  - This suggests that better items often include multiple plausible alternatives that partition weaker learners in different ways, useful both educationally (feedback reveals misconception type) and psychometrically (better separation).
5. Use option-length balance as a quality-control step, not as the main design goal
  - Given the weak association with discrimination after adjustment, option-length metrics should flag items for review rather than define quality. If the correct option is much longer/shorter than others (e.g., outside ±1 SD or >25% longer than the median), check whether you have inadvertently introduced cueing or whether qualifiers (“most”, “usually”) are applied unevenly across options [3, 4].
6. Treat negative discrimination as a safety signal
  - Items with negative discrimination warrant urgent review for ambiguity, miskeying, or a distractor that could reasonably be argued as better than the keyed answer [3]. In Dental Materials, where terminology and definitions are nuanced, negative discrimination may also reflect imprecise wording or an overly broad stem.

This study used classical test theory (CTT) metrics because they are transparent, interpretable and commonly used for item-bank review in educational settings. However, CTT-derived item statistics are sample-dependent, meaning that facility and discrimination estimates partly reflect the characteristics of the examinee cohort as well as the item itself [19]. Item response theory (IRT) approaches, including Rasch or two-parameter logistic models, can provide item parameters less dependent on the specific examinee sample and may be valuable as DentTest response counts grow [20]. However, IRT requires stronger model assumptions and substantially larger samples; proposed minimum sample sizes in medical education are approximately 200 examinees for one-parameter models and 500 for two-parameter models [19]. The present consenting cohort of 76 users, distributed across a large item pool, was therefore considered insufficient for reliable IRT calibration.

### Limitations

This study uses observational, formative question-bank data and therefore supports inference about item behaviour within this online context rather than direct generalisation to summative examinations; interpretation of item statistics depends on intended use and validity evidence [21, 22]. The consenting cohort was small for item-analysis purposes: 76/134 eligible users provided research consent, and consenting users may not be representative of all DentTest users. Since non-consenting users were excluded before analysis, consent-related selection bias cannot be excluded. In addition, users engaged with DentTest through self-directed formative study and were not standardised with respect to stage of undergraduate training, prior Dental Materials knowledge, or the pattern and intensity of question-bank use. These learner- and usage-level differences may influence both facility and discrimination estimates and were not controlled for in the present analysis.

The modest per-item response counts contributed to low split-half stability of *D*; discrimination index values should therefore be treated as provisional item-review signals rather than stable item properties. Although aggregate associations across 691 items may identify useful item-bank patterns, individual item classifications should be re-evaluated as response counts grow. We mitigated part–whole contamination using leave-one-item-out ability ranking, but tertile-based discrimination indices remain sensitive to sampling variability at modest N.

Finally, we operationalised cueing using option character-length metrics; other cueing mechanisms (e.g., grammatical cues, absolute terms, patterned options) were not assessed and may still influence item performance [3, 18].

### Implications for educators and platform development

For Dental Materials educators, the findings support prioritising distractor quality and moderate facility when building SBA assessments. For digital platforms, automated flags such as “very easy + 0 functioning distractors,” “high cueing risk,” and “negative discrimination” can focus editorial time on the items most likely to benefit from revision, enabling continuous improvement of item banks.

## Conclusions

In this formative online question bank, Dental Materials SBA items with higher discrimination were associated with moderate facility, stronger distractor functioning and more informative distractor-choice patterns. Option-length cueing showed only a weak association with discrimination and was not independently associated after accounting for facility and distractor metrics, supporting its use as a quality-control flag rather than a primary design target. A data-informed item-writing and revision heuristic therefore emerges: aim for moderate facility, engineer ≥2 functioning distractors grounded in plausible alternatives, confirm informative wrong-answer patterns, and screen for negative discrimination and option-length cueing to prioritise revision.

## Data Availability

The full user-level response dataset cannot be made publicly available because it contains platform-derived learner analytics and links to proprietary question-bank content. An anonymised item-level summary dataset and analysis code may be made available from the corresponding author on reasonable request, subject to institutional governance and removal of any question text or potentially identifying information.
